# Yinchenhao Decoction Protects Against Acute Liver Injury in Mice With Biliary Acute Pancreatitis by Regulating the Gut Microflora–Bile Acids–Liver Axis

**DOI:** 10.1155/2024/8882667

**Published:** 2024-06-27

**Authors:** Xianlin Zhao, Xiajia Wu, Qian Hu, Jiaqi Yao, Yue Yang, Meihua Wan, Wenfu Tang

**Affiliations:** ^1^ West China Center of Excellence for Pancreatitis Institute of Integrated Traditional Chinese and Western Medicine West China Hospital Sichuan University, Chengdu 610041, China; ^2^ West China School of Medicine Sichuan University, Chengdu 610041, China; ^3^ Institute of Respiratory Health and Multimorbidity West China Hospital Sichuan University, Chengdu 610041, China

**Keywords:** acute liver injury, biliary acute pancreatitis, gut microflora–bile acid–liver axis, Yinchenhao decoction

## Abstract

**Background and Aims:** Acute liver injury (ALI) often follows biliary acute pancreatitis (BAP), but the exact cause and effective treatment are unknown. The aim of this study was to investigate the role of the gut microflora–bile acids–liver axis in BAP-ALI in mice and to assess the potential therapeutic effects of Yinchenhao decoction (YCHD), a traditional Chinese herbal medicine formula, on BAP-ALI.

**Methods:** Male C57BL/6 mice were allocated into three groups: negative control (NC), BAP model, and YCHD treatment groups. The severity of BAP-ALI, intrahepatic bile acid levels, and the gut microbiota were assessed 24 h after BAP-ALI induction in mice.

**Results:** Our findings demonstrated that treatment with YCHD significantly ameliorated the severity of BAP-ALI, as evidenced by the mitigation of hepatic histopathological changes and a reduction in liver serum enzyme levels. Moreover, YCHD alleviated intrahepatic cholestasis and modified the composition of bile acids, as indicated by a notable increase in conjugated bile acids. Additionally, 16S rDNA sequencing analysis of the gut microbiome revealed distinct alterations in the richness and composition of the microbiome in BAP-ALI mice compared to those in control mice. YCHD treatment effectively improved the intestinal flora disorders induced by BAP-ALI. Spearman's correlation analysis revealed a significant association between the distinct compositional characteristics of the intestinal microbiota and the intrahepatic bile acid concentration.

**Conclusions:** These findings imply a potential link between gut microbiota dysbiosis and intrahepatic cholestasis in BAP-ALI mice and suggest that YCHD treatment may confer protection against BAP-ALI via the gut microflora–bile acids–liver axis.

## 1. Introduction

Acute pancreatitis (AP), characterized by acinar cell injury and inflammation in pancreatic tissue, is one of the most common gastrointestinal illnesses requiring acute hospitalization [1]. It is estimated that 34 people per 100,000 have AP [2]. The etiology of AP is varied and complex; however, among these causes, biliary stones, alcoholism, and hyperlipidemia are the main causes [3, 4]. Specifically, biliary AP (BAP) is commonly caused by gallstones or bile acid reflux into the pancreatic duct [4, 5]. Acute liver injury (ALI) is one of the most common extrapancreatic complications of AP, particularly in patients with BAP [6]. The liver, the primary organ responsible for energy and substance metabolism, which involves protein synthesis, detoxification, and bile acid production, is prone to disease [7]. Thus, preventing and treating ALI in patients receiving BAP at an early stage is crucial. Unfortunately, treatment options for BAP-ALI remain limited due to a lack of knowledge about the exact mechanism and pathogenesis of this damage.

The roles of the gut-liver axis in liver disease have been increasingly explored in recent years [8, 9]. The gut-liver axis refers to the bidirectional relationship between the gut and its microbiota, as well as the liver, which results from the integration of signals generated by dietary, genetic, and environmental factors [9, 10]. Bile acids participate in the complex interaction between the liver and gut microbiota. Bile acids are produced in the liver and have direct and indirect antimicrobial effects; they influence the microbiota, which in turn regulates bile acid pool size and composition [8–10]. It has been noted that host-gut microbe interactions are perturbed in a variety of experimental liver diseases, and unprotected intestinal barriers fuel hepatic inflammation [10, 11]. Several studies in recent years have shown that intestinal flora changes during the development of AP, which can affect its severity [12, 13]. Therefore, bile acid metabolism and the gut microbiota could be potential drug targets for treating BAP-ALI via the microbiota–bile acid–liver axis.

Herbal medicines have been used in traditional Chinese medicine (TCM) for thousands of years, and they have considerable clinical experience in China. In recent years, herbal medicines have gained worldwide popularity due to their unique efficacy on multiple targets. Yinchenhao decoction (YCHD) from the classic medical book “Treatise on Febrile Diseases” (Han Dynasty in ancient China), composed of *Artemisia capillaris* Thunb (Herba Artemisiae Capillaris, Yinchenhao), *Gardenia jasminoides* Ellis (Fructus Gardeniae, Zhi-zi), and *Rheum officinale* Baill (Radix Rhei Officinalis, Da-huang), is a classic TCM formulation used to treat various liver diseases and was first described for the Treatment of Febrile Disease in AD 200 [14, 15]. With few side effects and low toxicity, YCHD is used as a complementary and alternative treatment for ameliorating clinical symptoms, improving liver function, reducing liver inflammation, and improving quality of life [15–17]. YCHD has been demonstrated to attenuate cholestasis, but its hepatoprotective mechanisms of action in BAP-ALI have not been elucidated [17–20]. Due to the microbiota–bile acid–liver axis, the gut microbiota composition profoundly affects bile acid metabolism [11, 21]. As reported by Perumpail et al., intestinal bifidobacterium species degrade bile salts and promote fecal bile acid excretion, which inhibits cholesterol absorption and enhances enterohepatic circulation [22].

Therefore, the aim of this study was to determine the role of the gut microbiota–bile acid–liver axis in BAP-ALI mice and the mechanism by which YCHD alleviates intrahepatic cholestasis caused by intestinal microbiota disorder and improves liver injury.

## 2. Materials and Methods

### 2.1. Animals

The experiments utilized young male C57BL/6 mice weighing approximately 22 ± 2 g and aged between 7 and 8 weeks. Sichuan University's Jiangan Laboratory Animal Center (Chengdu, China) provided the mice, which were reared under controlled conditions at 22 ± 2°C and 65 ± 10% humidity. In accordance with the approved animal study protocol (no. 20211469A) of Sichuan University's West China Hospital Animal Experiments Committee, animal experiments were carried out at West China Science and Technology Park. Throughout all the animal experiments, the Principles of Laboratory Animal Care (NIH publication no. 85Y23, revised 1996) were followed.

### 2.2. YCHD Preparation

The YCHD, composed of *Artemisia capillaris* Thunb (Tarragon, Yinchenhao), *Gardenia jasminoides* Ellis (Gardenia, Zhi-zi), and *Rheum officinale* Baill (Rhubarb, Da-huang) with a weight ratio of 3:1:2, was acquired from Chengdu University of TCM (Chengdu, China). According to the original proportions (3:2:1 in weight) of the Treatise on Febrile Diseases, the spray-dried powders of Yinchenhao (18 g), Zhi-zi (12 g), and Da-Huang (6 g) were combined and then reconstituted with sterile distilled water to approximately 1 g/mL before being orally administered to mice [23].

### 2.3. Study Design

Thirty male mice were randomly divided into three groups (*n* = 10 for each group): negative control (NC), BAP model (BAP), and YCHD-treated (YCHD) groups. Prior to the experiment, the animals were fasted with free access to water for 24 h after acclimation. BAP in mice was induced by retrograde infusion of 2% sodium taurocholate (Bailingwei Technology Co., Ltd., China) into the biliopancreatic duct at 0.6 mL/min with a microinfusion pump (1.0 mL·kg^−1^ BW) [24, 25]. For the same procedure, the mice in the NC group were given an equivalent amount of saline (0.9% NaCl) instead of sodium taurocholate. After recovering from anaesthesia, an oral dose of 1.0 mL/100 g body weight of freeze-dried YCHD powder was administered to the mice [23]. All the mice were sacrificed with 2% sodium pentobarbital (0.04 mg/g BW) 24 h after administration, and arterial blood, feces, liver, and pancreatic tissues were collected for analysis.

### 2.4. Serum Enzymes and Liver Enzyme Assays

The serum levels of amylase, alanine aminotransferase (ALT), aspartate aminotransferase (AST), and total bile acid (TBA) were measured using an automated blood biochemical analyser (Chemray 800, Rayto, China). The measurement of serum amylase is commonly used to diagnose AP. ALT, AST, and TBA are commonly used as liver injury markers in blood tests.

### 2.5. Bile Acid Analysis

The extraction and analysis of bile from liver tissue samples were performed using liquid chromatography tandem mass spectrometry (LC–MS/MS) [26]. The AB Sciex QTRAP 6500 LC–MS/MS platform was utilized to detect the bile acid content, and MetWare (http://www.metware.cn/) was the chosen tool. Briefly, 20 mg of samples were extracted using 200 *μ*L of methanol after being crushed in a ball mill. For quantification, 10 *μ*L of an internal standard (IS) mixture (1 *μ*g/mL) was included in the extract as an IS. The samples were placed in a freezer at a temperature of −20°C for 10 min to cause protein precipitation. After centrifuging for 10 min at 12,000 revolutions per minute at 4°C, the resulting liquid was transferred to fresh plastic microtubes. The samples were dried and then dissolved in 100 *μ*L of 50% methanol (*v*/*v*) for subsequent LC–MS analysis. The provided samples were examined utilizing an LC–ESI–MS/MS system (UHPLC, ExionLCTMAD, https://sciex.com.cn/; MS, Applied Biosystems 6500 Triple Quadrupole, https://sciex.com.cn/).

### 2.6. Intestinal Microbiota Analysis

The 16S rRNA gene was sequenced from the cecal contents to analyse the potential distribution and structural characteristics of the intestinal microbiota as described previously in detail at Rhonin Biosciences Co. (Rhonin Biotechnology Ltd., Chengdu, China) [27, 28]. In brief, using a commercial kit (Major Bio, Shanghai, China), bacterial DNA was extracted from the feces. Using broad-range bacterial primers (515F and 806R), total DNA was amplified and purified via agarose gel electrophoresis (2% *w*/*v* agarose) using a Zymoclean Gel DNA Recovery Kit (Zymo Research). A New England BioLabs NEBNext Ultra II DNA Library Prep Kit for Illumina was used to prepare the libraries. Sequencing was conducted with the HiSeq Rapid SBS Kit v2 (Illumina, FC-402-4023 500 Cycles) according to the instructions for the Illumina HiSeq 2500 platform (PE250). The QIIME platform v1.9.0 was used for subsequent data processing after quality control. Operational taxonomic units (OTUs) were clustered by Usearch (10.0.240, UPARSE-OTU algorithm, http://www.drive5.com/usearch/) with default parameters (97% identity). To analyse microbiological diversity, we used R software (v3.6.0) to analyse alpha and beta diversity. LEfSe analysis (https://bitbucket.org/biobakery/biobakery/wiki/Home) was used to compare species with significant differences among groups. The RDP classifier algorithm was used to categorize 16S rRNA gene sequences from the Silva (release 132) 16S rRNA database. STAMP version 2.1.3 was used to compare the intestinal microbiomes of the two groups. Using R software (4.1.2) and Cytoscape (3.9.0), we were able to compute and visualize the relationship between hepatic bile acids and the intestinal microbiota.

### 2.7. Hematoxylin and Eosin (H&E) Staining

The pancreas and liver tissues of the experimental mice were rinsed and weighed. We fixed pancreas and liver tissues in 4% formaldehyde overnight. Subsequently, the tissues were embedded in paraffin after being washed with water for 2 h. After the samples were sliced into uniform slices 2–4 mm thick, the sections were stained with H&E using standard methods. Then, the stained sections were observed and photographed. Two independent pathologists scored the histopathology (edema, neutrophil infiltration, necrosis, and hemorrhage) of the pancreas and liver [29, 30]. The histopathology score was calculated by averaging the combined scores assigned to each parameter by both investigators.

### 2.8. Statistical Analysis

All the experiments were independently repeated at least three times. The data obtained in our study were statistically analysed with GraphPad Prism (version 9) software (GraphPad Inc., USA). Continuous data are presented as the means and standard deviations (SDs). For multiple comparisons, one-way ANOVA and Tukey's multiple comparisons were used when the data had a normal distribution and homogeneity of variance. Mann–Whitney *U* tests were applied to compare the differences in the microbial *α* diversity indices (Chao1, PD, Shannon, and Simpson indices). A statistically significant correlation between the microbiota and bile acid concentration was estimated using Spearman's correlation analysis. *p* values less than 0.05 were considered to indicate statistical significance.

## 3. Results

### 3.1. YCHD Treatment Has a Therapeutic Effect on BAP-ALI

**Figure 1:**
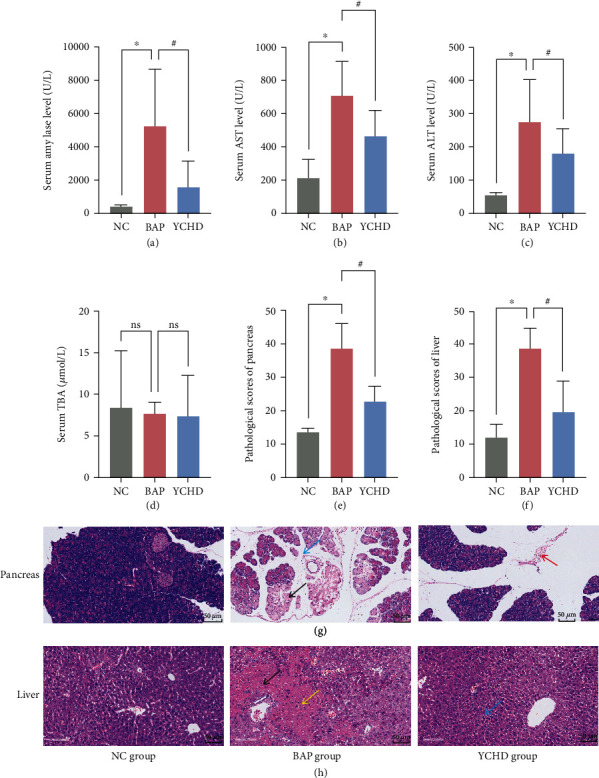
YCHD treatment has a therapeutic effect on BAP-ALI. (a–d) Serum amylase, AST, ALT, and TBA levels in the NC group, BAP model group, and YCHD treatment group (*n* = 10). (e, f) Histopathological scores of the pancreas and liver in each group (*n* = 4). (g, h) Morphological observation of the pancreas, liver, and gut using H&E staining (scale bar = 50 mm) in each group. All the experiments were independently performed at least three times. The data are expressed as means ± SDs with error bars. ^∗^*p* < 0.05 for NC vs. BAP; ^#^*p* < 0.05 for YCHD vs. BAP. ALT, alanine aminotransferase; AST, aspartate aminotransferase; BAP, biliary acute pancreatitis; NC, negative control; ns, not significantly different according to one-way ANOVA followed by Tukey's tests; TBA, total bile acid; YCHD, Yinchenhao decoction. Black arrow, necrotic cells; yellow arrow, inflammatory cell infiltration; red arrow, hemorrhage; blue arrow, edema cells.

The first step was to evaluate the effect of YCHD treatment on BAP-ALI. Amylase, ALT, AST, and TBA levels in the serum were measured and compared between the groups.  Figure 1 shows the experimental results. Compared to those of NC mice, BAP-treated mice had higher serum amylase, AST, and ALI levels, which were decreased by YCHD administration (Figures 1(a), 1(b), and 1(c), respectively) (*p* < 0.05). However, there were no statistically significant differences between the groups in terms of serum TBA levels (Figure 1(d)) (*p* > 0.05). Next, we examined the H&E-stained sections for signs of damage to the pancreas and liver. The histopathology damage in the BAP-ALI model group was significantly greater than that in the NC group (Figures 1(e) and 1(f)) (*p* < 0.05). The BAP group displayed marked cell edema, inflammation, necrosis, and hemorrhage in the pancreas and liver samples (Figures 1(g) and 1(h)). Treatment with YCHD reduced pancreatic and liver edema, inflammatory infiltration, and hemorrhagic hemorrhage (Figures 1(g) and 1(h)). These results suggested that the sodium taurocholate-induced BAP-ALI mouse model was successfully constructed and that treatment with YCHD had a therapeutic effect on BAP-ALI.

### 3.2. YCHD Treatment Alleviates Intrahepatic Cholestasis

**Figure 2:**
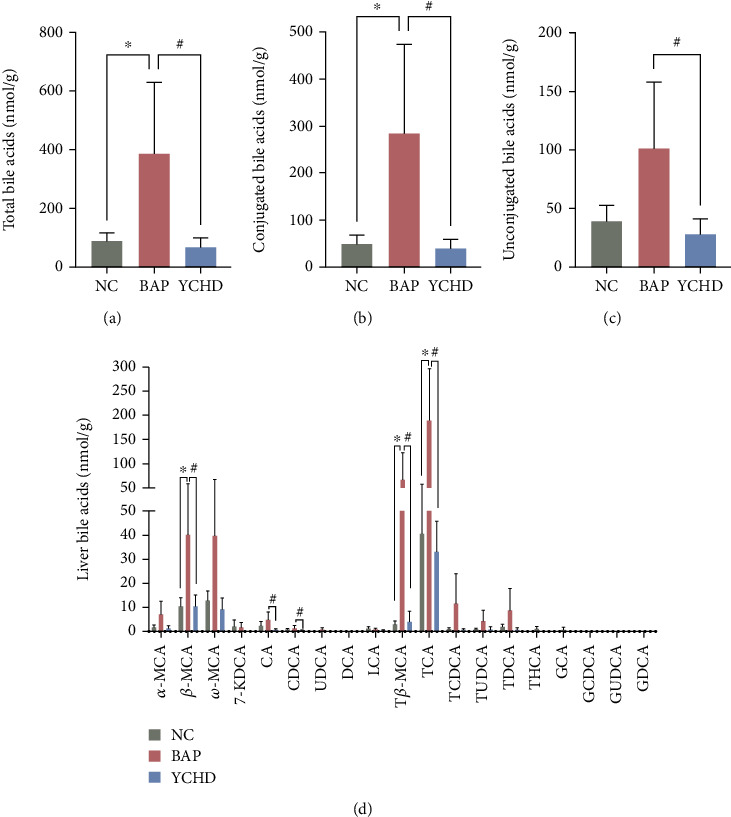
YCHD treatment alleviated intrahepatic cholestasis in BAP-ALI mice. (a–c) Concentrations of total bile acids, conjugated bile acids, and unconjugated bile acids in the hepatic tissues of each group (*n* = 4). (d) Composition of bile acids in the hepatic tissue of each group (*n* = 4). All the experiments were independently performed at least three times. The data are expressed as means ± SDs with error bars. ^∗^*p* < 0.05 for NC vs. BAP; ^#^*p* < 0.05 for YCHD vs. BAP; one-way ANOVA followed by Tukey's test. BAP, biliary acute pancreatitis; NC, negative control; YCHD, Yinchenhao decoction.

The composition and total concentration of bile acids in liver tissue from mice were determined using LC–MS/MS (Figure 2). Of the 50 bile acids used for screening, 19 were quantifiable and were included in the statistical analysis of hepatic and intestinal tissue. Figure 2 presents the experimental results. TBA levels in liver tissue were significantly greater in the BAP group than in the NC group but markedly lower in the YCHD group (Figure 2(a)) (*p* < 0.05). Further analysis revealed that the most prominent bile acid changes in liver tissue samples among the experimental groups were changes in conjugated bile acids, such as taurocholic acid (TCA), *β*-muricholic acid (*β*MCA), and tauro-*β*MCA (T-*β*MCA) (Figure 2(b)) (*p* < 0.05). These findings suggested that YCHD treatment could decrease the intrahepatic TBA concentration (mainly taurine-conjugated bile acids) in BAP mice.

### 3.3. YCHD Treatment Alters the Intestinal Microbial Composition

**Figure 3:**
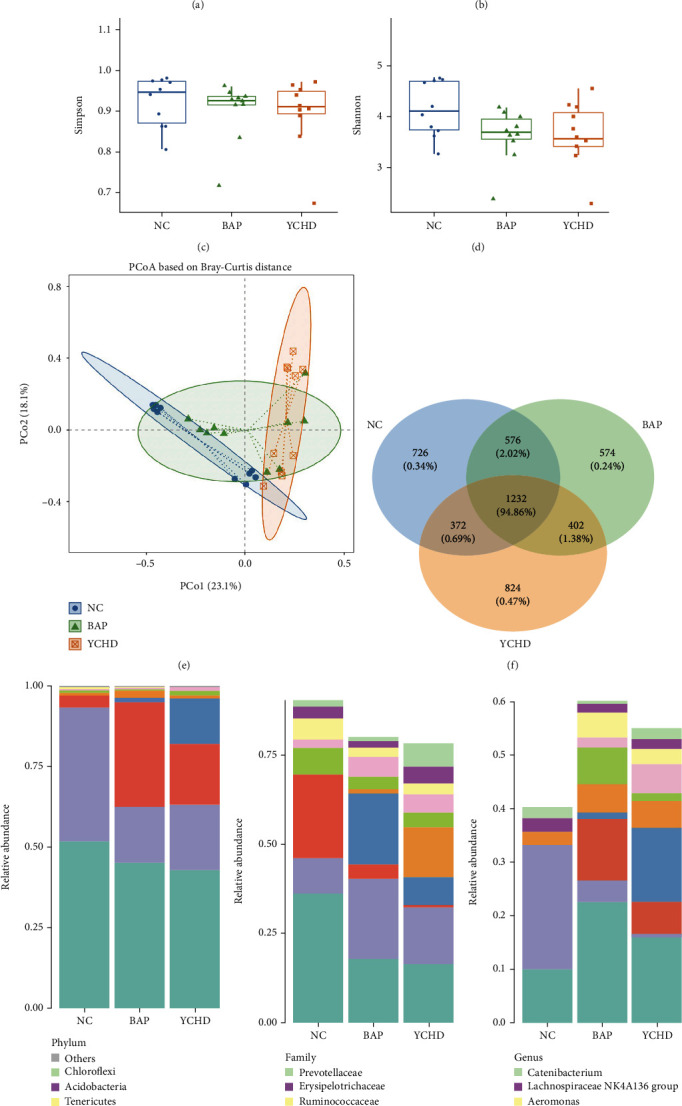
Analysis of the diversity and richness of the gut microbiome in mice. (a–d) Indices of Chao1, PD, Shannon, and Simpson in the NC group, BAP model group, and YCHD treatment group (*n* = 5), respectively. (e, f) Principal coordinate analysis (PCoA) and operational taxonomic units (OTUs) of the gut microbiome in each group (*n* = 5), respectively. (g–i) Relative abundance of differential microorganisms at the phylum, family, and genus levels in each group (*n* = 5), respectively. All the experiments were independently performed at least three times. ^∗^*p* < 0.05 for NC vs. BAP; ^#^*p* < 0.05 for YCHD vs. BAP according to Mann–Whitney *U* tests. BAP, biliary acute pancreatitis; NC, negative control; YCHD, Yinchenhao decoction.

To evaluate the impact of YCHD on the intestinal microbiota in BAP mice, fecal samples were analysed for the diversity and richness of gut bacteria (Figure 3). The Chao1, phylogenetic distance (PD), Shannon, and Simpson indices were used to determine the level of alpha diversity in the mice. The findings indicated that the BAP group had a significantly lower Chao1 index than the NC group (Figure 3(a)) (*p* < 0.05). After YCHD treatment, the above-year Chao1 index significantly improved (Figure 3(a)) (*p* < 0.05). The other levels of alpha diversity (PD, Shannon, and Simpson) did not significantly differ among the experimental groups, as shown in Figures 3(b), 3(c), and 3(d) (*p* > 0.05).

Then, we used principal coordinate analysis (PCoA) based on the Bray–Curtis distance to examine the beta diversity of the gut microbiota of the samples. As shown in Figure 3(e), the abscissas and ordinates represent the first and second principal coordinates, respectively, and the percentage represents each component's contribution to the sample difference. Blue, orange, and green represent the NC, BAP, and YCHD groups, respectively. The PCA score plots of the different groups overlapped with each other, which indicated that the BAP model and YCHD treatment significantly affected the beta diversity of the gut microbiota to some extent.

Based on the 16S rRNA gene sequence, OTUs were grouped based on a 97% clustering threshold. As shown in Figure 3(f), the Venn diagram of OTUs revealed that the colonic flora of each group of mice contained 1232 (94.86%) common OTUs. Additionally, each group had its own OTUs, including 726 (0.34%) in the NC group, 574 (0.24%) in the BAP group, and 824 (0.47%) in the YCHD group.

The distributions of the phyla, families, and genera and the proportions of microorganisms in the gut were analysed to further understand the changes in the intestinal flora, the results of which are shown in Figures 3(g), 3(h), and 3(i). Our results showed that the relative abundance of the microbiome changed significantly at the phylum, family, and genus levels. In particular, at the phylum level, compared to NC mice, BAP mice had greater relative abundances of Proteobacteria and Actinobacteria but lower relative abundances of Firmicutes and Bacteroidetes; YCHD treatment increased the relative abundance of Firmicutes and Verrucomicrobia and decreased the relative abundance of Proteobacteria and Actinobacteria in BAP mice (Figure 3(g)). At the family level, BAP stimulus decreased the relative abundance of Lactobacillaceae, Lachnospiraceae, Muribaculaceae, and Erysipelotrichaceae but increased the relative abundance of Enterobacteriaceae, Bacteroides, and Burkholderiales in mice. When compared with BAP mice, YCHD treatment could increase Prevotellaceae and *Akkermansia* relative abundances but decrease Lactobacillaceae, Enterobacteriaceae, and Bacteroidaceae relative abundances (Figure 3(h)). At the genus level, the relative abundances of *Bacteroides*, *Escherichia*, *Enterobacter*, *Enterococcus*, *Ralstonia*, and *Aeromonas* in the BAP group were greater than those in the NC group, while the relative abundance of *Lactobacillus* was significantly lower. Compared to those in the BAP group, the relative abundances of *Bacteroides*, *Escherichia*, *Shigella*, and *Enterobacter* were lower, while the relative abundances of *Akkermansia* and *Enterococcus* were greater (Figure 3(i)).

In summary, the intestinal flora of BAP model mice significantly changed, as observed above, while the imbalanced gut microbiota tended to recover after YCHD treatment.

### 3.4. There Is a Close Connection Between the Gut Microbiota and Intrahepatic Bile Acid in Mice

**Figure 4:**
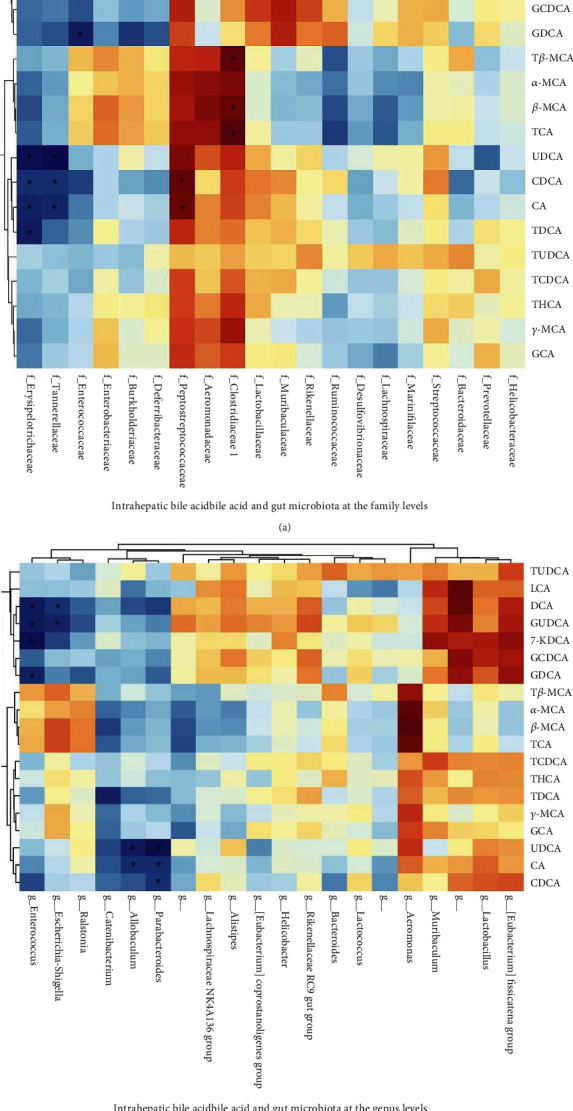
Spearman's correlation of the gut microbiota with intrahepatic bile acid in mice. (a) Heatmap of Spearman's correlations between intrahepatic bile acid and the gut microbiota at the family level. (b) Heatmap of Spearman's correlations between intrahepatic bile acid and the gut microbiota at the genus level. All the experiments were independently performed at least three times. The colours range from blue (negative correlation) to red (positive correlation). ^∗^*p* < 0.05.

The correlation of the gut microbiota and intrahepatic bile acid in mice was determined using Spearman's correlation analysis (Figure 4). Correlations that are negative are shown in blue, and correlations that are positive are shown in red. From the gut microbiota at the family level of view, strong positive associations were observed between (1) Clostridiaceae 1 and T-*β*MCA, *β*MCA, and TCA levels and (2) Peptostreptococcaceae and chenodeoxycholate (CDCA) and cholic acid (CA) levels in liver tissue, respectively (Figures 4(a)). Moreover, strong negative associations were observed between (1) Enterobacteriaceae and GUDCA level; (2) Erysipelotrichaceae and CA, glycoursodeoxycholic acid (GUDCA), 7-ketone deoxycholic acid (7-KDCA), and glycodeoxycholate (GDC) levels; (3) Tannerellaceae and ursodeoxycholic acid (UDCA), CDCA, and CA levels; and (4) Erysipelotrichaceae and UDCA, CDCA, CA, taurodeoxycholic acid (TDCA) levels in liver tissue, respectively (Figures 4(a)). At the genus level of gut microbiota, strong negative associations were observed between (a) *Parabacteroides* and UDCA, CA, and CDCA levels; (2) *Allobaculum* and UDCA and CA levels; (3) *Catenibacterium* and TDCA level; (4) *Escherichia* and *Shigella* and deoxycholic acid (DCA) and GUDCA levels; and (5) *Enterococcus* and DCA, GUDCA, 7-KDCA, and glycodeoxycholic acid (GDCA) levels in liver tissue, respectively (Figures 4(b)). The results revealed a significant correlation between the gut microbiota and intrahepatic bile acid in BAP-ALI mice.

## 4. Discussion

The classical Chinese medicine compound YCHD has a long history of clinical use in China for the treatment of liver diseases [15, 31]. However, the efficacy and underlying mechanisms of its use in treating BAP-ALI are unknown. Evidence suggests that cholestasis is relatively common in patients with BAP [32]. When cholestasis occurs, hepatocellular bile acid retention occurs, and excessive intrahepatic accumulation of bile acids can damage the liver [33]. However, how to improve cholestasis in BAP-ALI remains unclear. For more than 2000 years, YCHD has been recognized as a well-known TCM for treating cholestasis [18, 19]. In line with these reports, we also found increased levels of bile acids in the liver tissues of BAP-ALI mice in our study. Further research revealed that the most prominent bile acid change in liver tissue samples among the experimental groups was conjugated bile acids, such as TCA, *β*MCA, and T-*β*MCA. YCHD effectively reduced these levels in BAP-ALI mice. Moreover, YCHD reduced the levels of serum hepatic enzymes and hepatic damage according to histopathology. These results suggest that the increase in intrahepatic bile acid levels in BAP-ALI mice can likely be attributed to liver injury and that YCHD treatment has a therapeutic effect on BAP-ALI.

Recent studies have focused heavily on the intestinal microbiome in liver disease, but how this microbiome affects the liver has not been determined [9, 34]. Trillions of microorganisms reside in the gut microbiota. The gut microbiota coexists harmoniously with its host, participates in digestion and nutrient absorption, and helps maintain the integrity of the immune system to prevent pathogen colonization [9, 35]. Most of the intestinal flora is classified according to its natural attributes, such as Firmicutes, Bacteroidetes, Proteobacteria, Actinobacteria, Verrucomicrobia, Fusobacteria, and Cyanobacteria. Among the most common bacterial genera are *Bacteroides*, *Clostridium*, *Peptococcus*, *Bifidobacterium*, *Eubacterium*, *Ruminococcus*, *Enterococcus faecalis*, and *Peptostreptococcus* [36]. Additionally, the intestinal flora can be classified as mutually beneficial (*Bifidobacterium*, *Lactobacillus*, *Lactococcus*, etc.), conditionally pathogenic (*Escherichia coli*, *Enterococcus*, *Ruminococcus*, *Bacteroides*, etc.), and exclusively pathogenic (*Escherichia coli*, *Staphylococcus*, *Proteobacteria*, *Streptococcus*, *Peptostreptococcus*, etc.) [37, 38]. There is a strong correlation between the gut microbiome and a wide range of diseases, including gastrointestinal disorders, liver diseases, metabolic disorders, immunological disorders, and neurological disorders [35, 36]. The results of our study indicate that BAP-ALI mice had greater relative abundances of Proteobacteria and Actinobacteria at the phylum level; Enterobacteriaceae, Bacteroides, and Burkholderiales at the family level; and *Bacteroides*, *Escherichia*, *Enterobacter*, and *Enterococcus* at the genus level. Another important finding was that YCHD effectively reduced the relative abundances of Proteobacteria, Actinobacteria, Lactobacillaceae, Enterobacteriaceae, Bacteroidaceae, *Bacteroides*, *Escherichia*, *Shigella*, and *Enterobacter* but increased the relative abundances of Firmicutes, Verrucomicrobia, Prevotellaceae, *Akkermansia*, and *Enterococcus*. These results revealed that the structure of the gut flora of BAP-ALI mice was disrupted and that YCHD treatment improved the metabolic disorder of the intestinal flora to a certain extent. The alterations in the diversity of the intestinal microbiota are likely to impact intestinal function, particularly in relation to bile acid homeostasis in BAP. However, there is a limited understanding of the specific functional mechanisms involved in this process. Future research endeavors will involve the integration of metabolomic analyses to more comprehensively evaluate the functional alterations within the gut microbiota and their specific impacts on bile acid metabolism. This will encompass the investigation of metabolites such as short-chain fatty acids (SCFAs) and those associated with bile acid metabolism, as well as exploring bile acid receptor signaling pathways such as the farnesol X receptor (FXR) [33, 39].

The gut microbiota–bile acid–liver axis has recently gained global recognition [8, 10, 21]. Throughout development and life, the liver and intestines are intimately connected. A large portion of the liver's blood supply comes directly from the intestine, which conveys intestinal flora to the liver through its portal vein [40]. The gut microbiota–bile acid–liver axis refers to the physiological interactions between the gut and liver, such as the metabolism of bile acids and immunity to gut-derived signals (inflammatory) [21, 41]. Bile acids, as essential components of bile, possess antibacterial activity as well as physiological detergent properties. Bile acids and intestinal bacteria work together to regulate gastrointestinal homeostasis. The enteric microbiota and bile acids interact in two ways to regulate bile acid metabolism and microbiota composition during enterohepatic circulation [42]. It has been demonstrated that bacteria living in the intestine regulate bile acid production, synthesis, metabolism, and reabsorption, which in turn regulate the growth and populations of bacteria [42, 43]. Our study revealed that the gut flora of BAP-ALI mice was structurally disrupted, which was mainly characterized by a decrease in abundance and diversity, as well as changes in the structure of the flora. Moreover, cholestatic liver injury was discovered in BAP-ALI mice. The correlation of the gut microbiota and intrahepatic bile acid in mice was further analysed using Spearman's correlation analysis. Our study yields the following key results. Strong positive correlations were observed between Clostridiaceae 1 and T-*β*MCA, *β*MCA, and TCA levels and between Peptostreptococcaceae and CDCA and CA levels in liver tissue when considering the gut microbiota at the family level. Moreover, there were significant adverse associations between Enterobacteriaceae and GUDCA concentration; Erysipelotrichaceae and CA, GUDCA, 7-KDCA, and GDC concentrations; Tannerellaceae and UDCA, CDCA, and CA concentrations; and Erysipelotrichaceae and UDCA, CDCA, CA, and TDCA concentrations in the liver. Similar results have also been found at the genus level for the gut microbiota. The results above suggested an intricate interplay between bile acids and the gut microbiota in BAP-ALI mice. More importantly, our data highlight the protective effects of YCHD against cholestatic liver injury and gut flora imbalance. However, the specific mechanism of the relationship between bile acids and the gut microbiota requires additional study. As a bile acid-activated transcription factor, FXR maintains cholesterol homeostasis [44]. MCA and T-*β*MCA were identified as naturally occurring FXR antagonists produced by the gut microbiota [39, 45]. CDCA, which are primary free bile acids, were positively correlated with Lactobacillaceae, possibly because Lactobacillaceae contain bile salt hydrolase (BSH), which hydrolyses conjugated bile acids and increases free bile acid production [46]. The regulation of the intestinal-liver axis is mediated by FXR molecules, which play a role in modulating anti-inflammatory activity [47]. Future studies will aim to analyse FXR activity in liver tissue, elucidate the signaling pathways involved, assess the extent of hepatocyte apoptosis and necrosis, and investigate the regulatory effects of YCHD on BAP-ALI mice.

Effective animal models are crucial for investigating disease mechanisms and devising intervention tactics. The scientific community widely acknowledges that retrograde biliopancreatic duct infusion of sodium taurocholate can provoke BAP by mimicking the inflammatory processes linked to clinical BAP in humans [24, 25]. Bile acid plays a critical role in preserving homeostasis within the gut-liver axis [48]. The utilization of the sodium taurocholate–induced BAP model provides a more comprehensive representation of the effects of disruptions in bile acid metabolism and alterations in gut microbiota on hepatic function, thus supporting our research objective of exploring the gut-liver axis. It is imperative to identify the specific targets of YCHD in order to gain a more thorough understanding of its mechanism of action in BAP-ALI. Previous studies have demonstrated that a TCM network pharmacology approach can effectively elucidate the intricate interactions among multiple components and pathways targeted by the treatment [49]. In future studies, we plan to employ network pharmacology to forecast potential targets of YCHD components, utilize metabolomics to detect changes in gut microbiota and hepatic bile acid metabolites, and evaluate the regulatory impacts of YCHD components. This approach will enhance our understanding of YCHD's mechanisms of action.

## 5. Conclusions

**Figure 5:**
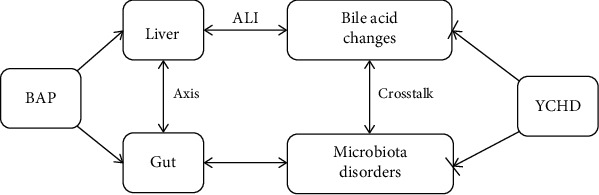
Schematic diagram of the mechanism of YCHD in treating BAP-ALI. ALI, acute liver injury; BAP, biliary acute pancreatitis; YCHD, Yinchenhao decoction.

The potential effects of YCHD on BAP-ALI mice were investigated first through the “gut microflora–bile acids–liver” axis. Based on these preliminary results, YCHD markedly attenuated BAP-ALI in mice by alleviating intrahepatic cholestasis, modulating bile acid composition, and shifting the intestinal microbiota composition. Moreover, a high degree of correlation between bile acids and the enteric microbiota was revealed in these experimental mice. According to the findings of this study, YCHD could ameliorate BAP-ALI in mice by regulating the interactions between the gut microbiota and intrahepatic cholestasis (Figure 5). However, the specific molecular mechanism through which the gut microbiota–bile acid axis regulates inflammation must be explored in animal models or cell lines.

## Data Availability

All relevant data can be made available for bona fide researchers on request from the authors.
